# *ATP1A3* mutation in rapid-onset dystonia parkinsonism: New data and genotype-phenotype correlation analysis

**DOI:** 10.3389/fnagi.2022.933893

**Published:** 2022-08-01

**Authors:** Lihua Yu, Guoping Peng, Yuan Yuan, Min Tang, Ping Liu, Xiaoyan Liu, Jie Ni, Yi Li, Caihong Ji, Ziqi Fan, Wenli Zhu, Benyan Luo, Qing Ke

**Affiliations:** Department of Neurology, The First Affiliated Hospital, Zhejiang University School of Medicine, Hangzhou, China

**Keywords:** *ATP1A3*, gene mutation, rapid-onset dystonia parkinsonism (RDP), genotype-phenotype correlation analysis, early diagnosis of RDP

## Abstract

**Background:**

Rapid-onset dystonia parkinsonism (RDP) is a rare disease caused by *ATP1A3* mutation with considerable clinical heterogeneity. Increased knowledge of RDP could be beneficial in its early diagnosis and treatment.

**Objective:**

This study aimed to summarize the gene mutation spectrum of *ATP1A3* associated with RDP, and to explore the correlation of *ATP1A3* variants with RDP clinical phenotypes.

**Methods:**

In this study, we reported two RDP patients from a family with a novel inherited *ATP1A3* variant. Then, we reviewed and analyzed the available literature in English focused on *ATP1A3*-causative RDP. A total of 35 articles covering 15 families (59 patients) and 36 sporadic RDP cases were included in our analysis.

**Results:**

The variant A813V (2438C>T) in *ATP1A3* found in our cases was a novel mutant. Delays in diagnosis were common, with a mean delay time of 14 years. *ATP1A3* had distinct RDP-related mutation hotspots, which consisted of exon8, 14, 17, and 18, and the most frequently occurring variants were T613M and I578S. Approximately 74.5% of patients have specific triggers before disease onset, and 82.1% of RDPs have stable symptoms within 1 month. The incidence rates of dystonia and bradykinesia are 100 and 88.1%, respectively. The onset site varied and exhibited a rostrocaudal gradient distribution pattern in 45% of patients with RDP. Approximately 63.6% of patients had mild improvement after receiving comprehensive interventions, especially in gait disturbance amelioration.

**Conclusion:**

In patients with acute and unexplained dystonia or bradykinesia, gene screening on *ATP1A3* should be timely performed. When a diagnosis has been made, treatments that may be effective are to be attempted. Our study would be helpful for the early diagnosis and treatment of *ATP1T3*-related RDP.

## Introduction

Rapid-onset dystonia-parkinsonism (RDP, *DYT12*) identified a quarter century ago is a rare disorder characterized by the abrupt onset of asymmetric dystonia and parkinsonism, with bradykinesia, gait instability, and prominent bulbar symptoms (Brashear et al., 1996; Heinzen et al., 2014). RDP episodes typically occur within hours of a triggering event, such as fevers, alcohol consumption, exercise, emotional stress, childbirth, or infections, and progress in a few hours to a week (Tarsy et al., 2010; Barbano et al., 2012). Subsequently, the clinical symptoms of most patients maintain relatively stable, and levodopa treatment shows only no or only a minimal benefit (Heinzen et al., 2014). Disease onset within age ranges from 9 months to 55 years has been previously reported (Brashear et al., 2007). The clinical manifestations of RDP are heterogeneous, and family members can have different clinical symptoms. Thus, RDP diagnostic delay is very common (Tan et al., 2015; Haq et al., 2019). Understanding the RDP phenotype profile is of great significance for its early diagnosis and treatment.

*ATP1A3*, as an autosomal dominant pathogenic gene, is the only causative gene for RDP (Brashear et al., 1996, 2007). It encodes a neuron-specific P-type Na+/K+ ATPase that is closely related to the sodium-coupled transport of a variety of organic and inorganic molecules, osmoregulation, and electrical excitability of nerves and muscles (Heinzen et al., 2014). *ATP1A3* variation is also the primary cause of alternating hemiplegia of childhood (AHC), Relapsing encephalopathy with cerebellar ataxia (RECA) (Dard et al., 2015; Biela et al., 2021), early onset epileptic encephalopathy (Paciorkowski et al., 2015; Schirinzi et al., 2018), and cerebellar ataxia, areflexia, pes cavus, optic atrophy, and sensorineural hearing loss (CAPOS) (Heinzen et al., 2012). The clinical phenotype of AHC is characterized by recurrent episodes of hemiplegia and dystonia alternating in laterality, and the onset age is usually before 18 months (Rosewich et al., 2012), which is quite different from RDP. In addition, there is a specific AHC/RDP intermediate phenotype, with clinical symptoms overlapping with those of AHC and RDP. This type often presents symptoms of AHC in the early stage of the disease, and as the disease progresses, the clinical manifestations and outcomes are similar to those of RDP (Termsarasab et al., 2015; Pereira et al., 2016). Until now, nearly 30 mutations of *ATP1A3* related to RDP and the AHC/RDP intermediate type have been identified.

In this study, we reported cases of two RDP patients from a family carrying a novel *ATP1A3* mutation. We reviewed all *ATP1A3*-related RDP and AHC/RDP intermediate patients reported in the previously published English literature. We then performed a genotype-phenotype correlation analysis using the collected data.

## Materials and methods

### Patient cohorts

Two patients, including the proband and her subject mother, were enrolled in our study. Both fulfill the clinical diagnostic criteria (Rosewich et al., 2017), received detailed medical history collection and physical examination, performed head MRI scanning, cardiac color Doppler ultrasound, lumbar puncture examinations, and collected peripheral blood for whole exome sequencing.

In addition, we searched all published English literature with the keywords “Rapid-onset dystonia parkinsonism”, or “RDP” and “ATP1A3” in Pubmed and Web of Science database, and then enrolled all clinically diagnosed as RDP or intermediate AHC/RDP. All patients included in this study met the clinical diagnostic criteria for RDP or intermediate AHC/RDP (Pereira et al., 2016; Haq et al., 2019). Through manual screening, we included a total of 35 English literature and our two cases, including 15 RDP families (total of 59 patients) and 36 sporadic patients, were performed secondary analysis. Among these 95 patients, 23 of them only had ATP1A3 mutation information, for which clinical data were not available, and further genotype–phenotype correlation analysis was not included. Ultimately, a total of 60 typical RDP patients and 12 intermediated RDP/AHC patients were included in our genotype-phenotype analysis.

This study was approved by the Ethics Committee of the First Affiliated Hospital, College of Medicine, Zhejiang University, China. Both of the patients in this study agreed to participate and provided written informed consent.

### Whole exome sequencing and Sanger sequencing

The proband was subjected to whole exome sequencing (WES) analysis to test point mutation or small deletion/insertion. The genetic testing was carried out at Running Gene Medical Laboratory (Beijing, China). Genomic DNA was extracted and tested according to the manufacturer's standard procedure as reported in our previous study (Yu et al., 2021). The potential variant found in WES was validated by the Sanger sequencing method. Then the variant found in the proband was tested in the subjected mother and asymptomatic father using the same method (Yu et al., 2021).

### Computational prediction of variant pathogenicity

We used PolyPhen-2 (https://genetics.bwh.harvard.edu/pph2/), MutPreds (http://mutpred.mutdb.org/#qform), PROVEAN (https://provean.jcvi.org/protein_batch_submit.php?species=human), programs to predict the effect of missense variants. Then, the Clinvar database, Pubmed database, and the Human Gene Mutation Database (HGMD) were used to screen mutations reported in previous studies. The variant detected in our study was compared in the gene database of normal healthy people, including the Chinese Millionome Database (CMDB) (https://cmdb.bgi.com/cmdb/), 1000 Genomes Project (https://www.internationalgenome.org/) (Sampson et al., 2016; Yu et al., 2021).

### Statistical analysis

We used the Shapiro–Wilk to test whether the age of the patient fits a Normal distribution and the Mann–Whitney U test was used to detect the age differences between subgroups. The Chi-square test was used to compare differences in rates. All the tests were performed with SPSS 26 software.

## Results

### Identification of a novel *ATP1A3* mutation in a familial RDP

An 18-year-old female (III-1, the proband) Chinese patient presented abruptly with dysphagia and speech difficulty after a headache. Within 2 days, she gradually developed right-upper limb bradykinesia and dystonia, and lower limb stiffness. One year later, after a cough, the dystonia and bradykinesia of the limbs were significantly aggravated. She had an abnormal gait and could not walk independently. Her symptoms did not progress and remained stable. She took levodopa/carbidopa and trihexyphenidyl for 6.5 months, but with no obvious effect. She had a positive family history, and her mother had similar symptoms. The patient reported no exposure to drugs, toxins, or an uneventful medical history of neurological disorders. At the age of 20, she visited our department. Given her symptoms, we refined a series of tests. Psychological assessments using HAMD and HAMA scales revealed that she had significant anxiety and mild depression. Cognitive function assessments with MMSE and MoCA scales showed normal scores, and 3T-cranial MRI findings were normal. Cerebrospinal fluid examination (CSF) showed decreased homovanillic acid level. Using whole exome sequencing (WES), an *ATP1A3* missense heterozygous mutation was detected and verified by Sanger sequencing. *ATP1A3* variant c.2438C>T, located in exon18, results in alanine at position 813 being replaced by valine (p.Ala813Val, A813V). This novel variant was not found in Pubmed, HGMD, and 1000 Genomes projects. Therefore, the patient was diagnosed with RDP. After 1 month of treatments with baclofen, coenzyme Q10, and rehabilitation exercises, partial gait improvement was noted.

**Figure 1:**
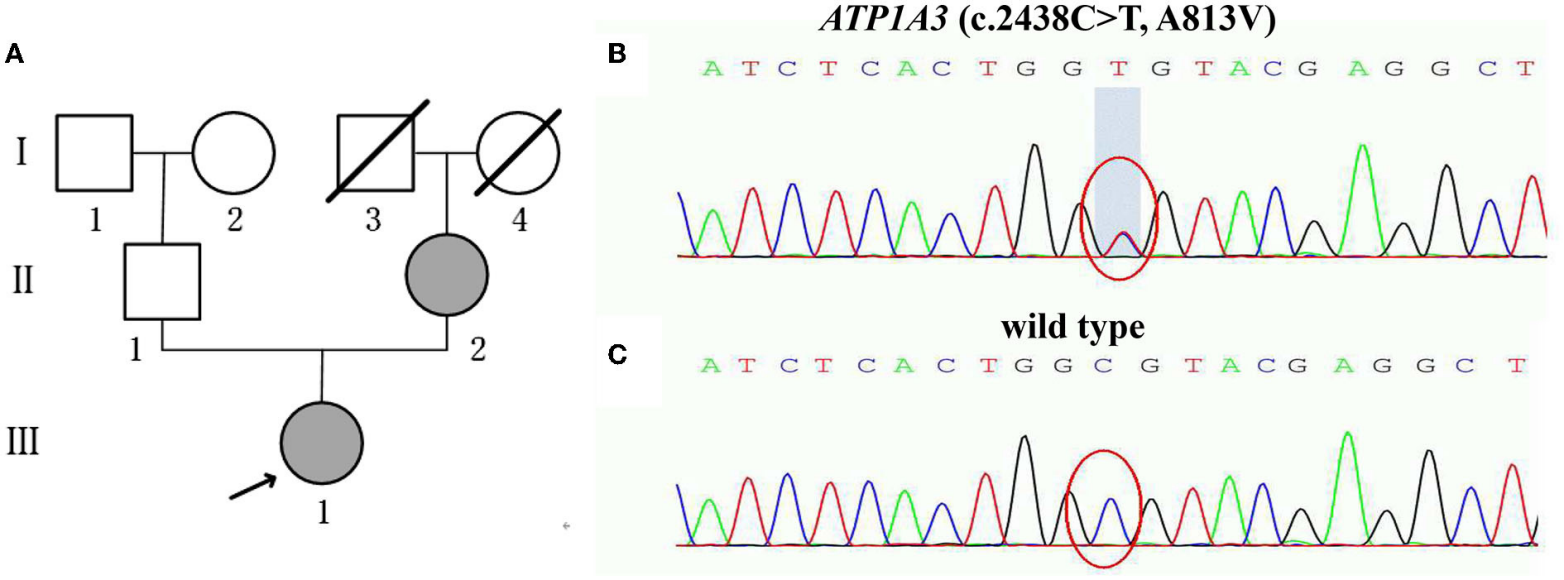
**(A)** Pedigree of the family with RDP showing the affected cases (fully shaded: indicate the ATP1A3 (c.2438C>T, A813V) mutation; **(B,C)** Sanger sequencing analysis of the mutation A813V; **(B)** Heterozygous p.A813V mutation carrier (the proband III-1 and the mother of the proband II-2); **(C)** Normal control (the father of the proband II-1).

Mother of the proband (II-2) showed the symptoms after she abruptly adopted an abnormal posture and unsteady gait following a strenuous exercise. Sanger sequencing confirmed that she carried the same *ATP1A3* c.2438C>T missense mutation. She did not receive any medication due to mild symptoms. Father of the proband (II-1) was completely normal and did not carry this variant (Figure 1).

### *ATP1A3* A813V was a potentially pathogenic mutant for RDP

The proband's manifestations met the clinical diagnostic criteria for typical RDP. The results suggested that *ATP1A3* variant alleles were co-segregated in the family. The mutation site is located in exon18 of *ATP1A3*, which constituted the mutation hotspot region. In the *ATP1A3* A813V variant, the original alanine was replaced by valine. As observed by crystallography, the side chain of valine was much larger than alanine, which changed the conformation of the ATP1A3 protein, thereby impairing its biological activity. The nature of the variant (A813V) was predicted to be highly disruptive, as indicated by a PolyPhen2 score of 0.663 (possibly damaging) and a SIFT-PROVEAN finding of deleterious (−2.846). In addition, the predicted result of MutPred2 was damage (0.575) and the predicted molecular mechanism was the loss of Helix. According to the criteria recommended by the ACMG grading guidelines criteria, the *ATP1A3* variant A813V was rated as a likely pathogenic mutant (Richards et al., 2015) (Supplementary figure 1). Taken altogether, variant A813V of *ATP1A3* potentially altered its function.

### Clinical and genetic spectrum of patients with *ATP1A3*-related RDP and intermediated AHC/RDP

A total of 95 patients with RDP and intermediate AHC/RDP, including 15 family and 36 sporadic patients with *ATP1A3* mutations, were enrolled in our study. The manifestations and genetic information of all enrolled patients are summarized in Supplementary table 1 (Pittock et al., 2000; Wunderlich et al., 2002; de Carvalho Aguiar et al., 2004; Zaremba et al., 2004; Kamphuis et al., 2006; Brashear et al., 2007, 2012; Lee et al., 2007; Kamm et al., 2008; Zanotti-Fregonara et al., 2008; Anselm et al., 2009; Blanco-Arias et al., 2009; Svetel et al., 2010; Tarsy et al., 2010; Barbano et al., 2012; Roubergue et al., 2013; Oblak et al., 2014; Rosewich et al., 2014a,b; Sasaki et al., 2014; Tan et al., 2015; Termsarasab et al., 2015; Wilcox et al., 2015; Liu et al., 2016; Nicita et al., 2016; Pereira et al., 2016; Sampson et al., 2016; Sweadner et al., 2016; Sousa et al., 2017; Wenzel et al., 2017; Marrodan et al., 2018; Haq et al., 2019; Boonsimma et al., 2020; Yuan et al., 2020; Hoshino et al., 2021; Nomura et al., 2021). Due to the lack of clinical data of some patients, a total of 60 patients with typical RDP and 12 patients with intermediate AHC/RDP were finally included for further analysis (Tables 1, 2) (Anselm et al., 2009; Brashear et al., 2012; Rosewich et al., 2014a; Sasaki et al., 2014; Termsarasab et al., 2015; Nicita et al., 2016; Pereira et al., 2016; Sousa et al., 2017; Boonsimma et al., 2020).

**Table 1:** Clinical characteristics of *ATP1A3*-related RDPs.

	**Total**	**Sporadic**	**Family**	***p*-value**
No.	60	22	38	
Sex (female)	36/60 (60%)	12/22 (54.5%)	24/38 (63.2%)	0.512
Onset age (y)*	21.2 ± 10.3	18.9 ± 6.9	22.5 ± 11.7	0.321
Diagnosis age (y)	35.2 ± 14.2	28.6 ± 8.7	39.1 ± 15.4	0.010
Triggers	38/51 (74.5%)	13/17 (76.5%)	25/34 (73.5%)	1.000
Time to stable ( ≤ 1 m)*	46/56 (82.1%)	14/22 (63.6%)	32/34 (94.1%)	0.015
Aymmetric onset	54/60 (90%)	18/22 (81.8%)	36/38 (94.7%)	0.179
Dystonia	60/60 (100%)	22/22 (100%)	38/38 (100%)	1
Parkinsonism	52/59 (88.1%)	21/22 (95.5%)	31/37 (83.8%)	0.240
F>A>L gradient*	27/60 (45%)	10/22 (45.5%)	17/38 (44.7%)	0.957
Onset site				0.854
Face (bulbar)	25/60 (41.5%)	10/22 (45.5%)	15/38 (39.5%)	
Arm/leg	25/60 (41.7%)	9/22 (40.9%)	16/38 (42.1%)	
≥2 sites	10/40 (25%)	3/22 (13.6%)	7/38 (18.4%)	
Bulbar symptoms	57/60 (95%)	22/22 (100%)	35/38 (92.1%)	0.292
Cognitive disorders	11/28 (39.3%)	2/10 (20%)	9/18 (50%)	0.226
Mental symptoms	25/34 (73.5%)	6/9 (66.7%)	19/25 (76%)	0.670
Clinical course				0.422
Improvement	25/53 (47.2%)	11/21 (52.4%)	14/32 (43.8%)	
Stable	25/53 (47.2%)	8/21 (38.1%)	17/32 (53.1%)	
Deterioration	3/53 (5.6%)	2/21 (9.5%)	1/32 (3.1%)	
Effective treatment	21/33 (63.6%)	7/16 (43.8%)	12/17 (70.6%)	0.166
Ethic				0.819
Europe	27/58 (46.6%)	9/19 (47.4%)	18/39 (46.2%)	
Asia	13/58 (22.4%)	5/19 (26.3%)	8/39 (20.5%)	
Others	18/58 (31.0%)	5/19 (26.3%)	13/39 (33.3%)	

* y = year, m = month; F>A>L gradient: face>arm>leg, indicates the symptoms with gradient progression from face to arm and then to the leg.

**Table 2:** Summary of the genetic and clinical features of the intermediate AHC/RDP patients.

**Reference**	**Sex^a^**	**Age**	**Onset age ^b^**	**Amino acid**	**First symptoms and onset**	**Present symptoms**	**Non-motor symptoms**	**Progression**	**Drug response**
Anselm et al. (2009)	M	4y	3y	D923N	Episodes of flaccidity and lack of motion	Bulbar symptoms, oromotor dystonia, apraxia	Mutism	IMP	–
Brashear et al. (2012)	F	1y	8m	R756H	Acute hypotonia and dysphagia	She could sit alone, but not crawl. could point and vocalize with no words	–^d^	IMP	NA
Sasaki et al. (2014)	M	4y	2y	D923N	Unable to ambulate, flaccid paralysis of left side	Dysarthria, drooling, ataxic gait, general mild hypotonia, and clumsiness	NA^c^	St	NA
Rosewich et al. (2014a)	F	15y	4.5y	G867D	Recurrent paroxysmal flaccid hemiplegia alternating	General performance was slow, moderate ataxia, mild dystonia oflimbs	Cognitive disorder	St	NA
Pereira et al. (2016)	M	5y	3y	G358D	Left brachial-predominant dystonia	Alternating hemiplegia, quadripledic episodes, dystonia, bulbar symptoms	Cognitive impairment	IMP	flunarizine
Termsarasab et al. (2015)	F	24y	early teens	E951K	Global developmental delay and alternating hemiplegic episodes	Risus sardonicus, dysarthria, drooling, asymmetric dystonic and parkinsonism	Cognitive disorder, stunting	IMP	Botulinum toxin
	F	10y	Childhood	D801N	Global developmental delay	Indistinct speech, hands dystonia, slow choreiform movements of fingers	Cognitive disorder, stunting	IMP	Levodopa
Nicita et al. (2016)	F	17y	3m	D583Y	Focal clonic seizures, paroxysmal episodes of weakness	Ideomotor slowdown, apathy, facial hypomimia, parkinsonism	Cognitive disorder, psychopathy	IMP	Lorazepam, trihexyphenidyl, flunarizine
	F	18y	11m	R756C	Episode of left hemiplegia	Generalized dystonia, hypophonia, bradykinesia and ataxic gait	–	IMP	Trihexyphenidyl
Sousa et al. (2017)	F	14y	14m	R756H	Myoclonus of the upper limbs, clumsy walking	Dystonia, cervical tilt, slight bradykinesia and generalized hyporeflexia	–	IMP	Levodopa
	M	49y	6y	R756H	Unable to walk, to swallow and to articulate words	Writing clumsy, slight dysarthria and facial and upper limb action dystonia	–	IMP	Levodopa
Boonsimma et al. (2020)	F	9m	2m	A809P	Developmental regression	Muscular hypotonia, dystonia	Cognitive disorder, Psychopathy	NA	NA

a Sex: F, female; M, male.

b d, day; w, week; m, month.

c NA, not available.

d “–” means no or negative.

### Systematic review of clinical features of RDP patients

A systematic review of the available published clinical data of RDP patients with *ATP1A3* mutations was conducted in this study. A total of 60 typical RDP, composed of 38 familial RDPs from 15 pedigrees and 22 unrelated sporadic RDPs, were included in our analysis (Table 1). Overall, the proportion of female patients (60%) was slightly higher than that of male patients (40%). The age at RDP onset was 21.2 ± 10.3 years (range 4–55 years), the diagnosis age was 35.2 ± 14.2 years, and the average diagnostic delay time was 14 years. About 74.5% (38/51) of the patients had definite triggers before the onset of RDP or before their symptoms significantly worsened. The triggers vary, and even the same patient met with different triggers. The most common triggering factors (over 80% of the reported triggers) included psychological stress, fever (heat), alcohol, exercise, head trauma, and childbirth. Other less frequently seen triggering factors were also reported, such as medicine (prochlorperazine) intake, mock air defense drill, anesthetic application, respiratory tract infection, travel sickness, and operation. Most patients experienced a rapid onset process, with 82.1% reaching a stable state within 30 days.

In terms of clinical symptoms, all patients had dystonia; 88.1% of them also presented with parkinsonism (mainly bradykinesia and muscle rigidity), and 90% of the patients experienced an asymmetric onset. A common rostrocaudal gradient [Face (Bulbar) > Arm > Leg] was presented, along with the characteristic manifestations that were found in 45% of RDP patients. The proportion of the patients with a limb (arm/leg) onset and face (bulbar) onset accounted for 41.5 and 41.7%, respectively. Bulbar symptoms were very common, appearing in 95% of the RDPs. Non-motor symptoms, such as cognitive disorders and mental symptoms, were not uncommon, especially the latter appearing in ~73.5% of the patients, whereas cognitive disorders occurred in 22.7% of patients. There was a lack of effective drugs for treating RDP, and RDP patients had poor responses to dopamine/carbidopa (Pittock et al., 2000; Rosewich et al., 2014b). In this study, comprehensive treatments showed partial efficacy in 63.6% of patients, with the most significantly improved symptom being gait disturbance. In terms of auxiliary examination, blood test and CT/MRI examination did not reveal specific changes, but severe brain atrophy was detected in one patient using cranial MRI (Sweadner et al., 2016). Molecular imaging tests, including PET-CT and SPECT, indicated some specific changes (Table 3) (Kamphuis et al., 2006; Zanotti-Fregonara et al., 2008; Anselm et al., 2009; Svetel et al., 2010; Tarsy et al., 2010). Decreased expression of homovanillic acid (HAV) in cerebrospinal fluid was found in RDP patients; Among eight patients who underwent the HAV test, three had a decreased HAV level. The potentially effective treatments included benzodiazepines (diazepam, or lorazepam), anti-parkinsonian drugs (levodopa/carbidopa, trihexyphenidyl, or amantadine), and other drugs such as baclofen, propranolol, a vitamin compound, and botulinum toxin injection. Of these, baclofen and trihexyphenidyl were primarily reported (Supplementary table 1). However, the current evidence regarding the efficacy of deep brain stimulation (DBS) treatment was very limited and controversial. In a previous study, the symptoms of one sporadic patient were not alleviated with DBS of bilateral globus pollidus internus (Gpi) (Deutschlander et al., 2005), whereas a modest benefit was achieved in another patient using DBS of the GPi (Kamm et al., 2008). Considering the clinical course of RDP, both improvement and stable state rates were 47.2%, and the deteriorating case was rare (5.6%). The results of previous functional studies on *ATP1A3*-related RDP, mainly involving the decreased activity of Na^+^-K^+^ ATPase and reduced affinity to cytoplasm Na^+^, were summarized in Table 4 (Rodacker et al., 2006; Blanco-Arias et al., 2009; Heinzen et al., 2012; Sweadner et al., 2016).

**Table 3:** Summary of molecular imaging findings on RDP and intermediate AHC/RDP.

**Reference**	**Mutation**	**Inspection method**	**Results**
Brashear et al. (1999)	D801Y	[^11^C]β-CFT PET	The volume of distribution was larger in the caudate and putamen;
Kamphuis et al. (2006)	I274T	1. FP-β-CIT-SPECT 2. IBZM-SPECT	1. Normal; 2. Reduce receptor D2 receptor density within the striatum on both sides.
Zanotti-Fregonara et al. (2008)	D923N	1. [123I]-FP-CIT 2. [99mTc]-HMPAO	1. Normal; 2. Normal.
Anselm et al. (2009)	D923N	FDG-PET	1.1 month after onset: moderate hapermetabolism in the striatum; 2. 8 years later: decrease metabolic activity in both thalami and the left putamen.
Svetel et al. (2010)	S684F	FP-CIT-PET	Nomal and symmetric uptake in both striatum
Tarsy et al. (2010)	G829A	PET	Mildly decreased metabolism in the bilateral thalamus and cerebellum.

**Table 4:** The functional findings of *ATP1A3* mutations on RDP and intermediate AHC/RDP.

**Mutation**	**Function of mutation**	**References**
T613M	Cause reduction of enzymatic affinity to cytoplasmic Na^+^; Alter Ca^2+^ homeostasis leading to cell damage.	(Rodacker et al., 2006)
1013Ydup	No defect in the biogenesis or plasma membrane targeting; Decrease survival in response to auabain challenge; Cause a 50-fold decrease in Na^+^ affinity.	(Blanco-Arias et al., 2009)
D923N	Do not reduce mRNA and protein expression of *ATP1A3*; Decrease the activity of the Na^+^/K^+^-ATPase.	(Heinzen et al., 2012)
G867D	Do not reduce in protein expression; A minor decrease of ATPase activity.	(Heinzen et al., 2012)
D801N	Do not reduce mRNA, mild reduce protein expression of *ATP1A3*; Decrease the activity of the Na+/K+-ATPase.	(Heinzen et al., 2012)
G316S	Impair pump acitivity; Partial inhibition of activity, often with reduced Na+ affinity.	(Sweadner et al., 2016)

Based on family history, we divided all RDP cases into two subgroups, sporadic and familial RDP. We compared their data and analyzed the difference in the clinical symptoms between the two groups. Of all clinical symptoms, only the diagnosis age showed a significant difference between the two groups (*p* = 0.010).

### Systematic review of clinical features of patients with intermediate AHC/RDP

A total of 12 patients diagnosed with intermediate AHC/RDP were included in our analysis, including 10 sporadic and 1 pedigree case. The male-to-female sex ratio was 1:2. The ages of these patients at onset were usually <5 years, with a mean diagnosis age of 13.49 ± 13.44 years, and a delay in diagnosis age was still very common. The majority of patients had definite triggers before disease onset, which was similar to RDP occurrence, and the most common triggering factors included fever, mental disorder, and exercise.

The onset symptoms and intermediate AHC/RDP vary greatly, manifesting as episodes of flaccidity, recurrent paroxysmal flaccid hemiplegia, limb dystonia or hypotonia of the limbs, myoclonus, and other non-motor symptoms such as seizure and global developmental delay. As the disease progressed, motor symptoms, including bulbar symptoms, dystonia, parkinsonism, and ataxic gait became increasingly common. In the early stage of intermediate AHC/RDP, the patients' clinical symptoms were similar to those of patients with AHC. With the progression of the disease, the phenotype of the intermediate AHC/RDP mimicked that of RDP. With regard to drug treatment, there was also a lack of effective treatments for intermediate AHC/RDP patients. Similarly with RDP treatment, partial effects might be achieved by administrating levodopa, trihexyphenidyl, lorazepam, and botulinum toxin injection. In addition, flunarizine was also reported to be potentially effective in two previous studies (Nicita et al., 2016; Pereira et al., 2016). In terms of outcome, the prognosis of patients with intermediate AHC/RDP was better than that of RDP patients, with ~9 of 12 patients showing improved condition (Table 2).

### Genotype-phenotype correlation analysis

**Figure 2:**
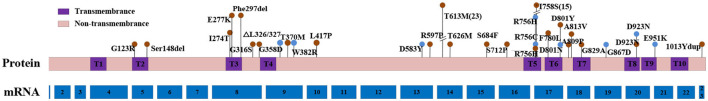
Schematic diagram of the location of RDP or intermediate AHC/RDP-causing mutations in ATP1A3 mRNA and protein. Red dots show RDP-causing mutations and blue dots dedicate intermediate AHC/RDP- causing mutations. The height of the dot represents the number of reported cases for this mutation, the shortest dot represents only one reported case for this mutation. The number of mRNA represent each corresponding exon. RDP, rapid-onset dystonia parkinsonism; AHC, alternating hemiplegia of childhood.

A total of 32 *ATP1A3* mutations have been reported in the literature, including 29 missense mutations and 3 nonsense mutations. Among them, 23 genetic mutations were only associated with the RDP phenotype, 7 were associated with the intermediate AHC/RDP phenotype, and the other 2 variants D923N and R756H were linked with RDP and intermediate AHC/RDP. Of all *ATP1A3* variants, T613M and I758S were predominant, with 25 and 15 RDP patients carrying these two variants, respectively. The mutational clusters were obvious, most of the mutations (87.5%) located in exons 8, 14, 17, and 18, with the corresponding areas being domain transmembrane regions (T3 and T5), and cytoplasmic regions (between T4 and T5). Approximately 58.3% of the mutations in the intermediate AHC/RDP mutational clusters are located in exons 9 and 17 (Figure 2). Most *ATP1A3* mutants were associated with a single disease phenotype, including RDP, AHC, or intermediate AHC/RDP, while a small portion of the mutants was correlated with different phenotypes. For example, *ATP1A3* D923N and R756H could present RDP or intermediate AHC/RDP phenotype (Zanotti-Fregonara et al., 2008; Anselm et al., 2009; Brashear et al., 2012; Tan et al., 2015), while *ATP1A3* variants 1013Ydul, E277K, D923N were linked with the phenotype of RDP or AHC (Termsarasab et al., 2015).

In previously reported RDP families, patients carrying the same mutation usually showed similar clinical manifestations and pathological changes. Oblak et al. (2014) reported three RDP patients from the same family with *ATP1A3* I758S mutant who had analogous clinical manifestations, including similar triggers, cognitive disorder, and severe psychotic disorder, the pathological changes of them were also comparable. In another RDP family with *ATP1A3* Ser148del variant, all nine patients were female, and the only male carrier did not show any symptoms (Wilcox et al., 2015). Barbano et al. (2012) reported an RDP family with *ATP1A3* T613M mutant, four subjects not only had the same onset and symptoms, but also show similar non-motor symptoms, including cognitive disorder with predominant verbal fluency impairment, and mood disorder.

## Discussion

In this study, we reported a Chinese RDP family carrying a novel *ATP1A3* variant 2438C>T (A813V), which was predicted to be a potential pathogenic mutation. Our case provided a genetic marker for the diagnosis and treatment of RDP, the discovery of this variant also expanded the existing knowledge on the genetic spectrum of RDP. We included all English literature on RDP and intermediate AHC/RDP, and a systematic review and genotype-phenotype correlation analysis were performed on the clinical and genetic findings of 60 RPD patients and 12 patients with intermediate AHC/RDP (Tables 1, 2). The following major results were found: (1) RDP diagnosis delay was common; (2) There was evidence supporting a genotype-phenotype correlation in RDP; (3) *ATP1A3*-related RDP and intermediate AHC/RDP might be different phenotypes of the same disease, also called a spectrum disorder; (4) The recognition of onset symptoms and timely genetic screening on *ATP1A3* mutations facilitated early diagnosis of RDP; (5) There were no significant differences exist in the clinical manifestations between family and sporadic patients.

Diagnosis delay is very common for *ATP1A3*-related RDP and intermediate AHC/RDP. We found that the diagnosis age was delayed by an average of about 14 years compared with the onset age. This phenomenon was more pronounced in the RDP family, where the diagnosis age and diagnosis delays were 39.1 ± 15.4 years and 16.6 years, respectively, much higher than those of sporadic RDP patients. Thus, patients can lose their opportunity for early treatment, and the disease burden on patients and their families would be increased. Several reasons may contribute to this finding: (1) Incomplete penetrance of *ATP1A3* is very common, the symptoms of the parents of the proband may be milder, more atypical, and even asymptomatic, resulting in some patients being diagnosed at an older age; (2) In the early period when the disease of RDP was recognized for family patients, the genetic diagnosis technologies at that time were not advanced, which also might lead to misdiagnosis or diagnosis delay. (3) As a rare disease, the clinical manifestations of RDP are heterogeneous. Some neurologists may not have sufficient knowledge about this disease. (4) The most important reason may be that the previous clinical diagnosis criteria were too strict, such as seeking a clear rostrocaudal gradient (F>A>L) or features of Parkinsonism (Brashear et al., 2007). In our statistical analysis, rostrocaldal gradient accounted for only 45%, whereas the rate of parkinsonism was 88%. Therefore, based on the development of genetic diagnostic technologies, we suggest the following changes in the diagnosis criteria: (I) unexplained acute onset; (II) dystonia (especially with prominent bulbar findings); (III) with or without bradykinesia; (IV) with *ATP1A3* variant. Some other symptoms such as clear trigger presence before onset, a clear rostrocaudal gradient, onset age of more than 18 months, the onset-to-stable time within 1 month, no or minimal responses to levodopa, and non-motor symptoms (psychiatric symptoms and cognitive dysfunction) support the diagnosis of RDP (Haq et al., 2019).

Genotype-phenotype correlation constitutes an important aspect of RDP pathogenesis. The predominant *ATP1A3* mutation locations in RDP patients were in exons 8, 14, 17, and 18 (a rate of 87.5%), the corresponding functional areas were domain transmembrane regions (T3 and T5) and the cytoplasmic region between T4 and T5, which was different from the reported common mutation regions in exon 5, 7, 8, 9, 16, 20, 21, and 22 of *ATP1A3* in AHC (Rosewich et al., 2014b). About 58.3% of the mutations in intermediate AHC/RDP mutational clusters are located in exons 9 and 17. The most common *ATP1A3* mutants were T613M and I758S, which were found in 42.1% of all RDP patients. The majority of patients with T613M mutant came from Europe, and the founder effect may be a plausible explanation. Patients carrying the same *ATP1A3* mutation tend to exhibit similar clinical phenotypes, including not only similar motor symptoms, but also non-motor symptoms, such as cognitive impairment, psychotic disorder, and verbal fluency impairment (Barbano et al., 2012; Oblak et al., 2014; Wilcox et al., 2015). We expect this correlation to become stronger as more *ATP1A3*-related RDP cases are reported. Meanwhile, the clinical heterogeneity of *ATP1A3*-related RDPS was significant, and the same mutation could be associated with different clinical phenotypes. The phenotypes of the patients with D923N mutant were presented as AHC, RDP, or intermediate AHC/RDP (Zanotti-Fregonara et al., 2008; Anselm et al., 2009; Roubergue et al., 2013), patients with R756H mutant could manifest as RDP or intermediate AHC/RDP phenotype (Brashear et al., 2012; Tan et al., 2015; Nicita et al., 2016). Additionally, *ATP1A3* E277K and 1013Ydul mutants were reported to be associated with RDP or AHC phenotype (Blanco-Arias et al., 2009; Brashear et al., 2012; Termsarasab et al., 2015). The reason for this phenomenon may be that *ATP1A3*-related diseases may be a spectrum of diseases. In addition to mutations in *ATP1A3*, other factors such as environmental factors and the interaction with other genes may also contribute to the pathogenesis of RDP (Oblak et al., 2014).

Currently, extensive research has been conducted on *ATP1A3* mutations that may be a pathogenic cause for RDP or intermediate AHC/RDP. The reported variants included *ATP1A3* T613M, 1013Ydup, D293N, G867D, and D801N (Table 4). The mutations of *ATP1A3* don't result in reduced expression of the ATP1A3 mRNA and protein, mainly causing decreased activity of Na^+^-K^+^ ATPase, lowering its affinity to cytoplasmic Na^+^ (Rodacker et al., 2006; Blanco-Arias et al., 2009; Sweadner et al., 2016). Similar findings are shown for the effects of *ATP1A3* mutations in AHC (Heinzen et al., 2012). Based on this evidence, improving ATPase activity and affinity for Na^+^ may be an important direction for the treatment of ATP1A3-related disease.

Until now, *ATP1A3* is the only discovered pathogenic gene for RDP, AHC, intermediate AHC/RDP and CAPOS (Brashear et al., 2007; Heinzen et al., 2012). If a patient has suspicious clinical manifestations, timely *ATP1A3* screening is conducive to the early diagnosis of ATP1A3-related diseases. In addition, the identification of gene mutation is also the basis for genetic counseling and future gene modification therapy. In addition to genetic screening, some other auxiliary examinations may also facilitate the early and effective diagnosis of RDP. Downregulated expression of homovanillic acid (HAV) in CSF was found in 37.5% (3/8) of reported RDP patients. In addition, some studies have established that RDP patients also show specific PET and SPECT molecular imaging findings (Table 3). Therefore, when the clinical symptoms of patients are atypical or genetic screening cannot be performed, the HAV test of CSF and molecular imaging can provide more evidence for RDP diagnosis.

There is a lack of effective treatments for RDP and intermediate RDP/AHC, and patients exhibit almost no responses to levodopa. However, other treatments may be minimally effective, such as trihexyphenidyl, lorazepam, baclofen, flunarizine, Botulinum toxin injection, and rehabilitation therapy (Supplementary table 1). Therefore, for patients diagnosed with RDP and intermediate AHC/RDP, the above treatments should be recommended. Nevertheless, in our study, ~47.2% of RDP and 75% of AHC/RDP patients achieved partial improvements, especially showing ameliorated gait impairment (Table 1). Deep brain stimulation (DBS) therapy is a promising but controversial treatment for RDP. In the past, two RDP patients were reported to undergo DBS surgery in GPi. The therapy was completely ineffective for one patient, while the other patient showed partial improvements in the motor symptoms (Deutschlander et al., 2005; Kamm et al., 2008). However, future advancements in surgical methods and changes in intervention targets may enable breakthroughs in the current treatment status in this field.

Based on our analysis, we make the following recommendations for the diagnosis and treatment of patients with RDP. For patients with acute and unexplained dystonia or bradykinesia, gene screening on *ATP1A3* should be timely performed. When a diagnosis has been made, treatments that may be effective are to be attempted. We believe that the most potentially effective treatments in the future will be the following: (1) genetic modification therapy of *ATP1A3*; (2) drugs that improve the NA^+^-K^+^ ATPase activity; and (3) DBS surgery. The current understanding of RDP is still very limited. Further research on the clinical manifestations, the functions of the mutations in *ATP1A3*, and the discovery and development of effective treatments are necessary.

## Data availability statement

The datasets presented in this study can be found in online repositories. The names of the repository/repositories and accession number(s) can be found in the article/Supplementary material.

## Ethics statement

The studies involving human participants were reviewed and approved by the Ethics Committee of the First Affiliated Hospital, College of Medicine, Zhejiang University, China. The patients/participants provided their written informed consent to participate in this study.

## Author contributions

LY: design, analysis, and writing. GP: statistical analysis. YY: diagnosis of the cases. MT: collect case information. PL: literature query. XL: document analysis. JN: reference retrieval. CJ: drawing of the tables. ZF: drawing of the figures. WZ: literature screening. BL: editing of final version of the manuscript. QK: design and editing of final version of the manuscript. All authors contributed to the article and approved the submitted version.

## Funding

This work was supported by the Natural Science Foundation of Zhejiang Province (LY20H170003, LY), the National Natural Science Foundation (81471300, LY); the National Key R&D Program of China (2020YFB1313501, QK), the Natural Science Foundation of Zhejiang Province (LY21H090010, QK), the National Natural Science Foundation (82001287, WZ), and the National Natural Science Foundation of China (82101251, PL).

## Conflict of interest

The authors declare that the research was conducted in the absence of any commercial or financial relationships that could be construed as a potential conflict of interest.

## Publisher's note

All claims expressed in this article are solely those of the authors and do not necessarily represent those of their affiliated organizations, or those of the publisher, the editors and the reviewers. Any product that may be evaluated in this article, or claim that may be made by its manufacturer, is not guaranteed or endorsed by the publisher.
