# Supplementary figures and images for: ATP1A3 mutation in rapid-onset dystonia parkinsonism: New data and genotype-phenotype correlation analysis
Source: Front Aging Neurosci. 2022 Aug 1;14:933893. doi: 10.3389/fnagi.2022.933893 (PMC9376385; doi:10.3389/fnagi.2022.933893)

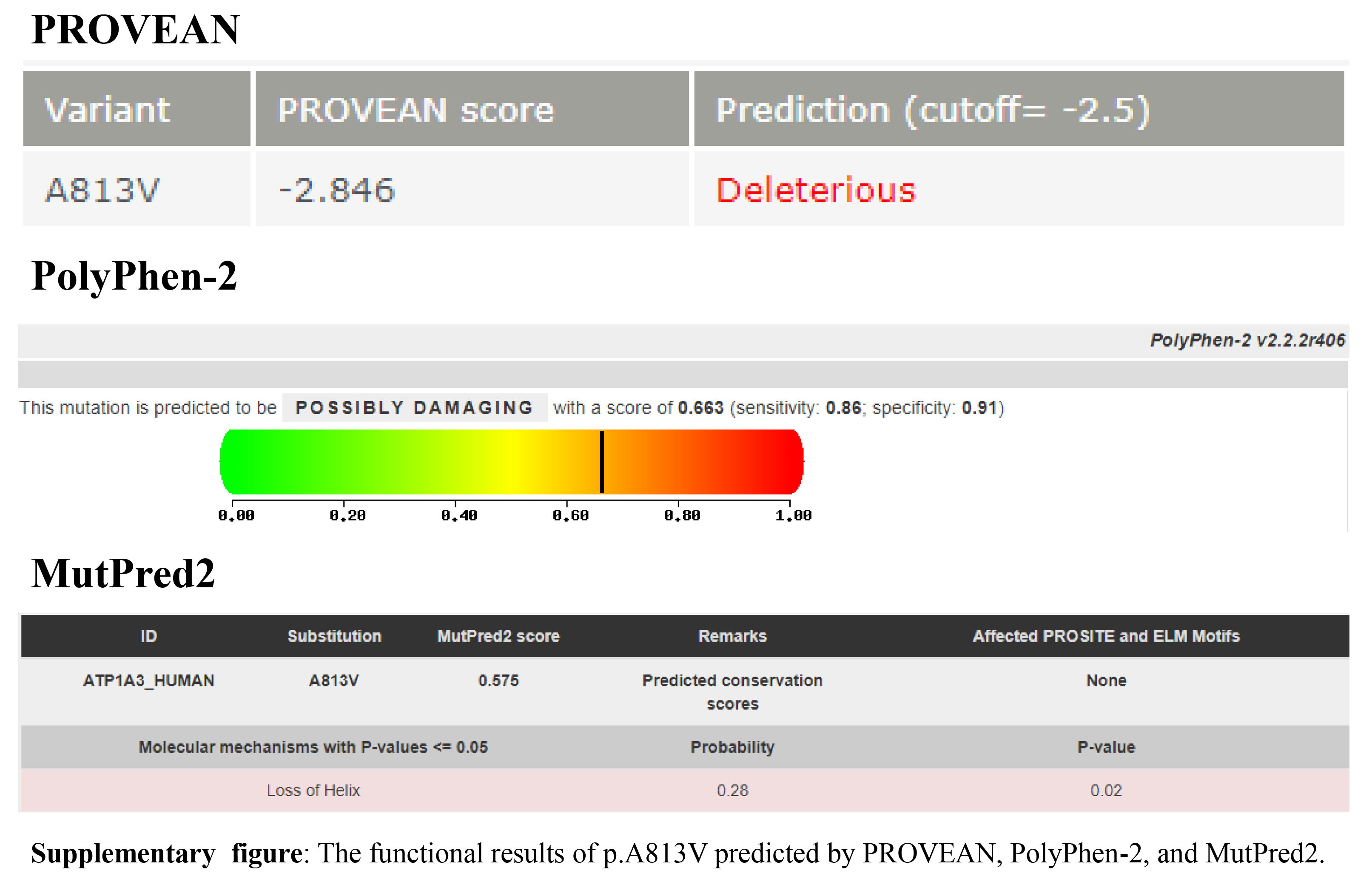

Supplement: Supplementary file 2 [file Image_1.TIF]
